# Author Correction: Integrated genomics point to immune vulnerabilities in pleural mesothelioma

**DOI:** 10.1038/s41598-022-08800-1

**Published:** 2022-03-21

**Authors:** Anca Nastase, Amit Mandal, Shir Kiong Lu, Hima Anbunathan, Deborah Morris‑Rosendahl, Yu Zhi Zhang, Xiao‑Ming Sun, Spyridon Gennatas, Robert C. Rintoul, Matthew Edwards, Alex Bowman, Tatyana Chernova, Tim Benepal, Eric Lim, Anthony Newman Taylor, Andrew G. Nicholson, Sanjay Popat, Anne E. Willis, Marion MacFarlane, Mark Lathrop, Anne M. Bowcock, Miriam F. Moffatt, William O. C. M. Cookson

**Affiliations:** 1 National Heart and Lung Institute, Imperial College London, Dovehouse Street, London, SW36LY UK; 2 Clinical Genetics and Genomics, Royal Brompton and Harefield NHS Foundation Trust, London, UK; 3 Department of Histopathology, Royal Brompton and Harefield NHS Foundation Trust, London, UK; 4 Medical Research Council Toxicology Unit, University of Cambridge, Cambridge, UK; 5 Department of Thoracic Oncology, Papworth Hospital, Cambridge, UK; 6 Department of Oncology, University of Cambridge, Cambridge, UK; 7 Department of Oncology, St George’s Healthcare NHS Foundation Trust, London, UK; 8 Department of Thoracic Surgery, Royal Brompton and Harefield NHS Foundation Trust, London, UK; 9 Department of Medicine, Royal Marsden Hospital, London, UK; 10 The Institute of Cancer Research, London, UK; 11 Department of Human Genetics, McGill Genome Centre, Montreal, QC Canada

Correction to: *Scientific Reports* 10.1038/s41598-021-98414-w, published online 27 September 2021

The original version of this Article contained an error.

In the Results section, under subheading ‘High level of VISTA is frequent in epithelioid mesothelioma and its expression level correlates with Hedgehog and immune pathway components’,

“Ki67 correlated with copy number burden (*P* = 0.03, r = 0.42) and with MTAP score (*P* = 0.04, r = 0.39), consistent with disordered cellular division accompanying *CDKN2A* deletion.”

now reads:

“Ki67 correlated with copy number burden (*P* = 0.03, r = 0.42) and with MTAP score (*P* = 0.04, r = − 0.39), consistent with disordered cellular division accompanying *CDKN2A* deletion.”

The original Article has been corrected.

