# Supplementary material for: Integrated genomics point to immune vulnerabilities in pleural mesothelioma
Source: Sci Rep. 2021 Sep 27;11:19138. doi: 10.1038/s41598-021-98414-w (PMC8476593; doi:10.1038/s41598-021-98414-w)
Supplement: Supplementary file 1 — Supplementary Information 1. [file 41598_2021_98414_MOESM1_ESM.pdf]

Integrated genomics point to immune vulnerabilities in pleural mesothelioma

SUPPLEMENTARY DATA

Anca Nastase[1¶], Amit Mandal[1¶], Shir Kiong Lu[1], Hima Anbunathan[1], Deborah Morris-Rosendahl[1,2], Yu Zhi Zhang[1,3], Xiao-Ming Sun[4], Spyridon Gennatas[1], Robert C Rintoul[5,6], Matthew Edwards[2], Alex Bowman[3], Tatyana Chernova[4], Tim Benepal[7], Eric Lim[8], Anthony Newman Taylor[1], Andrew G Nicholson[1,3], Sanjay Popat[9,10], Anne E Willis[4], Marion MacFarlane[4], Mark Lathrop[11], Anne M Bowcock[1], Miriam F Moffatt[1,*], William OCM Cookson[1,*]

Affiliations

1 National Centre for Mesothelioma Research, Imperial College London, UK

2 Clinical Genetics and Genomics, Royal Brompton and Harefield NHS Foundation Trust, London, UK

3 Department of Histopathology, Royal Brompton and Harefield NHS Foundation Trust, London, UK

4 Medical Research Council Toxicology Unit, University of Cambridge, Cambridge, UK

5 Department of Thoracic Oncology, Papworth Hospital, Cambridge, UK

6 Department of Oncology, University of Cambridge, Cambridge, UK

7 Department of Oncology, St George's Healthcare NHS Foundation Trust, London, UK

8 Department of Thoracic Surgery, Royal Brompton and Harefield Foundation Trust, London, UK

9 Department of Medicine, Royal Marsden Hospital, London, UK

10 The Institute of Cancer Research, London, UK

11 Department of Human Genetics, McGill Génome Centre, Québec, Canada

¶ Contributed equally to this study *Contributed equally to this study

*Corresponding authors: Professor William OCM Cookson: National Heart and Lung Institute, Dovehouse Street, London SW36LY, w.cookson@imperial.ac.uk +447788628503; Professor Miriam F Moffatt: m.moffatt@imperial.ac.uk

Contents

Supplementary Materials and Methods 3
Supplementary_Table 1. Targeted capture next-generation sequencing gene panel 4
Supplementary_Table 2. Patient demographics 5
Supplementary_Table 3. Significant copy number deletions and amplifications 6
Supplementary_Table 4. Genes differentially regulated in histological subtypes 7
Supplementary_Table 5. Correlation of *SUFU* and immune function genes and replication 8
Supplementary_Table 6. Genes differentially up-regulated in *BAP1* mutated tumours 9
Supplementary_Figure 1. Analytical structure 10
Supplementary_Figure 2. Oncoplot from targeted sequencing panel 11
Supplementary_Figure 3. Mutation characteristics 12
Supplementary_Figure 4. WES and RNA sequencing correlations for *RASSF7*, *RB1* and *SUFU* in primary and replication datasets 13
Supplementary_Figure 5. Oncoplot for WGS of mesothelioma primary cells 14
Supplementary_Figure 6. Effect of mutations in DNA damage repair related genes 15
Supplementary_Figure 7. Response of mesothelioma primary cells to tested drugs. 16
Supplementary_Figure 8. Original, uncropped and unprocessed blot images 17

Supplementary Materials and Methods

Visualisation of copy number results in UCSC Genome Browser

To visualise the copy number result through UCSC Genome Browser, patient-level copy number segments (Supplementary File 2_Table5B) with 'seg.mean' values >=0.3 or, <= -0.3 were selected. These segments were then converted to a file format that allows display of continuous value data as GenomeBrowser tracks: bedGraph format, using 'bedtools genomecov' (v.2.27). The output bedGraph file was then uploaded on UCSC GenomeBrowser (GRCh37/hg19 version). The visualisation is in the form of histograms with auto-scaling dependent on the genomic region selected for visualisation in the browser, and hence the number of CN segments contained within those genomic boundaries.

Strategy for filtering germline variants

Variants (whether from somatic calling or from unpaired tumour samples) were annotated using Ensembl Variant Effect Predictor (ver.92; GRCh37) with additional annotation resource of population level frequencies from gnomAD genomes and exomes (rel.2.0.1) and the ExAC (Exome Aggregation Consortium) non-TCGA (rel.0.3.1) resources. Variants present in the population databases with frequencies $>=10^{-3}$ were filtered out. Further filtering was done based on gene annotation to select only High or Moderate impact (i.e. protein-sequence altering) variants, or if the variant was predicted to be splice-altering (as per dbscSNV: database of splicing-consensus region SNVs). Further prioritisation of SNV candidates was done based on predicted deleteriousness in at least two of three algorithms: SIFT (ver.5.2.2), Polyphen (ver.2.2.2), and MutationTaster (online tool).
From our in-house studies on tumour samples with paired germline available, the above battery of filtering steps remove ~90% of germline variants. Thus, we are positive that we have been able to remove most of the germline variants in case of the unpaired tumour samples in our cohort.

Characterisation of the primary cells used for drug testing

Meso-27T, Meso-33T and Meso-70T used for drug testing were obtained from the MRC Toxicology Unit, University of Cambridge, UK. These primary cells were established as described in Chernova, T. *et al.* (ref 12). All preliminary analysis testing for tumour cell purity, morphology monitoring, cell lines authentication by STR DNA profiling and matching to the original tissue was performed prior to our study and detailed in Chernova, T. *et al.* (ref 12). Briefly, the authors have used differential trypsinisation with 0.05 and 0.1% trypsin to remove fibroblasts and select mesothelial cells, obtaining a mesothelioma primary cells purity of 97-100%. In our study, we have limited the number of passages to retain the initial characteristics of the primary cells.

Western blot

Cells were lysed with RIPA buffer in the presence of freshly added protease inhibitor and PhoSTOP (Roche). Cell lysate were centrifuged at 14000 rpm for 15 min, loaded on gel and transferred, after separated on SDS-PAGE gel, to nitrocellulose membranes. Membranes were blocked in PBS with 5% skim milk and 0.1% Tween-20, probed with antibodies to RB (Cell Signalling Technology, 9309), BAP1 (Santa Cruz, sc28383), Merlin (AbCam, ab88957), PTCH2 (Cell Signalling Technology, 2464), SUFU (Cell Signalling Technology, 2522), p16 (AbCam, ab108349) and beta-actin (AbCam, ab6276). Secondary antibodies used: goat-anti-mouse (ThermoScientific, 31320), AP conjugate and goat-anti-rabbit (ThermoScientific, 656122) AP conjugate.

Supplementary_Table 1. Targeted capture next-generation sequencing gene panel

ANKLE1	*CDKN2A*	*FOXD1*	*MAML3*	*NF2*	*SETD1B*	*TJP2*	*ZNF880*
APC	*CFAP45*	*HERC1*	*MLH1*	*NRAS*	*SETD2*	*TJP3*	
ASS1	*CREBBP*	*HERC2*	*MMP17*	*PIK3CA*	*SETD6*	*TP53*	
ATXN2	*DDX3X*	*ICA1*	*MSH2*	*PRDM12*	*SETDB1*	*TRAF7*	
BAP1	*DDX51*	*INADL*	*MSH3*	*PRKRA*	*SF3B1*	*ULK2*	
BRCA2	*EGFR*	*LATS1*	*MSH5*	*PTEN*	*SLC2A3*	*VEZF1*	
BRD4	*EP400*	*LATS2*	*MSH6*	*RB1*	*SYNE1*	*WDR89*	
CD163	*FBXW7*	*MACF1*	*NCOR2*	*RYR2*	*TET1*	*ZNF77*	

Supplementary_Table 2. Patient demographics

	Group					
	**Whole Exome Sequencing (n=50)**	**SNP genotyping (n=118)**	**Targeted Capture Sequencing (n=119)**	**RNA sequencing (n=35)**	**IHC (n=28)**	**All (n=121)**
**Gender (n, %)**						
M	39 (78.0)	103 (87.3)	103 (86.6)	25 (75.8)	23 (82.1)	105 (86.8)
F	11 (22.0)	15 (12.7)	16 (13.4)	8 (24.2)	5 (17.9)	16 (13.2)
M:F	3.5:1	6.9:1	6.4:1	3.13:1	4.6:1	6.6:1
**Age**						
Average ± SD	69.7 ± 8.2	72.1 ±8.5	72.1 ± 8.5	70.06 ± 8.3	67.6 ± 8.1	72.1 ± 8.4
median [range]	69.7 [52-90]	73.0 [51-90]	72.6 [51-90]	69.0 [55-90]	67 [52-82]	73 [51-90]
**Histology (n, %)**						
Epithelioid	34 (68.0)	89 (75.4)	88 (74.0)	26 (74.3)	19 (67.9)	90 (74.4)
Biphasic	14 (28.0)	23 (19.5)	25 (21.0)	6 (17.1)	9 (32.1)	25 (20.6)
Sarcomatoid	2 (4.0)	6 (5.1)	6 (5.0)	3 (8.6)	0 (0.0)	6 (5.0)
**Overall survival**						
Data available	92.0%	91.5%	92.4%	85.7%	100.0%	
All (months, [rate at 12 months])	9.93 [42.63%]	9.93 [42.24%]	9.93 [42.39%]	10.43 [43.75%]	12.8 [59.74%]	9.93 [42.0%]
Epithelioid	10.43 [45.16%]	11.6 [48.75%]	11.6 [48.75%]	12.27 [52.17%]	12.96 [68.42]	11.6 [48.15%]
Biphasic	10.86 [42.73%]	7.4 [29.54%]	7.4 [31.41%]	7.07 [33.33%]	10.87 [38.1%]	7.4 [31.41%]
Sarcomatoid	1.5 [0.0%]	3.66 [0.0%]	3.66 [0.0%]	1.5 [0.0%]	NA	3.67 [0.0%]
**Asbestos exposure (n, %)**						
Yes	32 (64.0)	63 (68.5)	63 (67.7)	20 (57.1)	21 (75.0)	83 (68.6)
No	16 (32.0)	27 (29.3)	28 (30.1)	11 (31.4)	6 (21.4)	36 (29.8)
Unknown	2 (4.0)	2 (2.2)	2 (2.2)	4 (11.4)	1 (3.6)	2 (1.6)

Supplementary_Table 3. Significant copy number deletions and amplifications

DELETIONS					
**cytoband**	**q value**	**residual q**	**wide peak boundaries**	**Kb**	**Principal Genes**
**9p21.3**	2.4E-112	2.4E-112	chr9:21927328-22031004	104	*CDKN2A, CDKN2B, C9orf53*
**3p21.1**	7.3E-10	7.3E-10	chr3:52433746-52449046	15	*BAP1*
**10q23.31**	8.6E-05	1.6E-04	chr10:91440057-91589280	149	*KIF20B, FLJ37201*
**16p13.3**	1.6E-04	1.4E-03	chr16:5128784-7773249	2644	*RBFOX1, FAM86A*
**4q13.3**	2.2E-03	3.2E-03	chr4:70932306-71062425	130	*CSN1S2AP, C4orf40, CSN1S2BP*
**6q14.2**	5.8E-03	5.5E-03	chr6:84563880-84759646	196	*CYB5R4*
**11p15.5**	1.6E-02	1.6E-02	chr11:906709-1083236	177	*AP2A2, MUC6*
**10q24.32**	2.5E-03	2.7E-02	chr10:104245589-104485300	240	*ARL3, SUFU, TRIM8*
**1p22.1**	7.5E-03	3.9E-02	chr1:93620394-93787866	167	*CCDC18*
**13q14.2**	4.2E-02	4.2E-02	chr13:48835311-49063155	228	*RB1, LPAR6*
**11q23.2**	4.7E-02	4.9E-02	chr11:113236976-113280927	44	*ANKK1*
**14q22.3**	4.8E-02	4.9E-02	chr14:56023823-56271005	247	*KTN1, RPL13AP3, LINC00520, KTN1-AS1*
AMPLIFICATIONS					
**cytoband**	**q value**	**residual q**	**wide peak boundaries**	**Kb**	**Principal Genes**
**11p15.5**	6.4E-48	6.4E-48	chr11:552679-587371	35	*RASSF7, PHRF1, LRRC56, LOC143666, C11orf35, MIR210, MIR210HG*
**19q13.43**	6.1E-45	6.1E-45	chr19:56572845-56674547	102	*ZNF444, NLRP5, ZNF787*
**5q35.2**	3.8E-39	1.2E-37	chr5:176025071-176043332	18	*GPRIN1, CDHR2*
**5q35.3**	1.8E-10	8.3E-08	chr5:179214819-179249072	34	*LTC4S, SQSTM1, MGAT4B, MIR1229*
**2p24.1**	9.1E-06	9.1E-06	chr2:21210344-21227220	17	*APOB*
**19p13.11**	9.1E-06	9.1E-06	chr19:17339697-17370189	30	*NR2F6, OCEL1, USHBP1*
**7p22.3**	1.2E-04	1.2E-04	chr7:2552807-2579466	27	*LFNG, BRAT1, MIR4648*
**16p13.3**	1.2E-04	1.2E-04	chr16:772127-786320	14	*NARFL, FAM173A, HAGHL, CCDC78*
**2q36.3**	1.6E-04	1.6E-04	chr2:227646960-227686906	40	*IRS1*
**16q22.1**	2.7E-04	2.7E-04	chr16:67220742-67229485	9	*E2F4, EXOC3L1*
**6p22.1**	6.9E-04	6.9E-04	chr6:29311440-29325601	14	*OR5V1*
**5q13.2**	1.5E-06	4.7E-03	chr5:70806522-70855825	49	*BDP1*
**11q21**	1.8E-02	1.8E-02	chr11:95569326-95603081	34	*MTMR2*
**6q23.3**	2.2E-02	2.2E-02	chr6:136582550-136648626	66	*BCLAF1*
**19p13.3**	2.2E-02	2.2E-02	chr19:1457003-1482901	26	*APC2, PCSK4, C19orf25*
**16q24.2**	4.6E-02	4.6E-02	chr16:88498491-89347394	849	*APRT, CBFA2T3, CDH15, CYBA, GALNS, MVD, PIEZO1, IL17C, ANKRD11, TRAPPC2L, CDT1, ZNF469, RNF166, ZC3H18, SLC22A31, ZFPM1, MGC23284, ZNF778, ACSF3, LINC00304, SNAI3, CTU2, PABPN1L, LOC400558, MIR4722*

Supplementary_Table 4. Genes differentially regulated in histological subtypes

Log2 Fold Change	Average Expression	Adjusted P Value	Chr	Name	Description
**5.23**	-2.62	2.10E-04	7	*NFE4*	Nuclear factor in erythrogenesis
**4.72**	-0.21	1.90E-04	3	*RP4-555D20.2*	lncRNA
**4.65**	-1.60	9.13E-04	17	*IGF2BP1*	Novel interacting partner of p38 MAPK. RNA-binding protein, involved in tumour progression
**4.50**	-0.31	9.13E-04	5	*GDNF*	Glial cell derived neurotrophic factor
**3.66**	1.57	9.92E-04	18	*CCBE1*	High levels contribute to aggressiveness and poor prognosis of Colon Cancer
**3.57**	3.41	7.26E-04	12	*WNT5B*	Activator of WNT signalling
**3.45**	-2.82	9.13E-04	15	*CTD-2033D15.2*	lncRNA
**3.33**	-3.95	9.91E-04	3	*RP4-555D20.4*	lncRNA
**2.89**	-3.88	7.18E-04	X	*RP11-320G24.1*	lncRNA
**2.66**	1.29	7.26E-04	17	*HS3ST3A1*	Tumour regulator and prognostic marker in breast cancer.
**2.27**	5.55	2.10E-04	1	*NAV1*	Potentiates migration of breast cancer cells
**2.25**	3.73	7.26E-04	2	*CHN1*	Actin dynamics in cell migration
**2.15**	3.41	7.26E-04	15	*GPR176*	Orphan G-protein-coupled receptor that sets the pace of circadian behaviour
**1.95**	6.25	2.58E-04	4	*SEPT11*	Septin 11
**1.73**	5.81	9.57E-04	6	*TRAM2*	Putative metastatic factor for oral cancer
**1.50**	3.88	9.57E-04	2	*SERTAD2*	Promotes oncogenesis in nude mice and is frequently overexpressed in multiple human tumours.

Genes differentially upregulated in sarcomatoid and mixed histology compared to epithelioid. *P* values are adjusted for multiple comparisons (false discovery rate <0.05).

Supplementary_Table 5. Correlation of *SUFU* and immune function genes and replication

NCMR n=35	*SUFU*		*PTCH2*		*PTCH1*		*CR1*		*KLRD1*		*PD-L1*	
PTCH2	-0.38	*2.5E-02*										
PTCH1	-0.37	*3.1E-02*	0.75	*1.7E-07*								
CR1	0.50	*2.2E-03*	-0.59	*1.8E-04*	-0.41	*1.4E-02*						
KLRD1	0.37	*2.9E-02*	-0.60	*1.4E-04*	-0.52	*1.3E-03*	0.56	*5.1E-04*				
**PD-L1**	0.10	*5.8E-01*	-0.39	*2.2E-02*	-0.44	*8.1E-03*	0.35	*4.1E-02*	0.37	*2.8E-02*		
**VISTA**	0.61	*1.1E-04*	-0.52	*1.3E-03*	-0.41	*1.3E-02*	0.43	*1.0E-02*	0.68	*7.5E-06*	0.09	*6.1E-01*
TGCA-Meso n=86												
PTCH2	-0.08	*4.6E-01*										
PTCH1	0.09	*3.9E-01*	0.77	*2.2E-16*								
CR1	-0.04	*7.1E-01*	-0.09	*4.1E-01*	-0.11	*3.4E-01*						
KLRD1	-0.04	*7.0E-01*	-0.36	*6.2E-04*	-0.33	*1.9E-03*	0.41	*7.9E-05*				
**PD-L1**	-0.26	*1.7E-02*	-0.22	*4.0E-02*	-0.30	*5.5E-03*	0.35	*8.1E-04*	0.40	*1.7E-04*		
**VISTA**	0.25	*2.1E-02*	-0.47	*5.9E-06*	-0.25	*2.2E-02*	-0.06	*5.9E-01*	0.37	*4.8E-04*	-0.09	*4.0E-01*
Bueno *et al*. n=211												
PTCH2	-0.22	*1.4E-03*										
PTCH1	-0.16	*1.8E-02*	0.71	*2.2E-16*								
CR1	0.30	*9.6E-06*	-0.20	*2.9E-03*	-0.23	*7.9E-04*						
KLRD1	0.26	*1.1E-04*	-0.37	*3.1E-08*	-0.35	*2.4E-07*	0.49	*7.1E-14*				
**PD-L1**	-0.08	*2.3E-01*	-0.16	*1.7E-02*	-0.31	*6.0E-06*	0.42	*2.8E-10*	0.38	*1.2E-08*		
**VISTA**	0.27	*5.6E-05*	-0.36	*6.0E-08*	-0.27	*8.3E-05*	0.06	*3.9E-01*	0.36	*6.8E-08*	-0.06	*3.5E-01*
Combined studies												
PTCH2	-0.21	*9.96E-05*										
PTCH1	-0.14	*1.20E-02*	0.73	*<2.2E-16*								
CR1	0.16	*3.53E-03*	-0.17	*2.23E-03*	-0.15	*6.87E-03*						
KLRD1	0.14	*9.81E-03*	-0.33	*6.71E-10*	-0.28	*3.33E-07*	0.55	*<2.2E-16*				
**PD-L1**	-0.09	*8.96E-02*	-0.18	*7.87E-04*	-0.28	*3.31E-07*	0.42	*1.78E-15*	0.42	*1.33E-15*		
**VISTA**	0.33	*6.93E-10*	-0.42	*2.00E-15*	-0.32	*3.81E-09*	-0.06	*2.66E-01*	0.22	*5.59E-05*	-0.11	*5.50E-02*

Pearson correlations between abundances of Hedgehog pathway transcripts *SUFU*, *PTCH1*, *PTCH2* and transcripts related to immune checkpoints. The official gene names for PD-L1 and VISTA are *CD274* and *VSIR*, respectively. Results are shown for the present study, two previous investigations and for all studies combined. Two sided-*P* values are shown in italics throughout.

Supplementary_Table 6. Genes differentially up-regulated in *BAP1* mutated tumours

Log2 Fold Change	Average Expression	Adjusted P Value	Chr	Name	Description
**6.09**	4.33	1.88E-02	20	*NNAT*	Marker of poor outcome in many cancers
**3.60**	1.47	7.77E-03	16	*NECAB2*	Neuronal calcium-binding protein that binds to adenosine A(2A) and metabotropic glutamate type 5 receptors
**3.20**	3.51	4.77E-02	22	*TAFA5*	Postulated to function as brain-specific chemokine or neurokine
**2.70**	2.43	1.88E-02	21	*PDE9A*	Phosphodiesterase 9A
**2.36**	1.68	4.77E-02	10	*RET*	Tyrosine kinase receptor leading to oncogenic signalling that is targetable with anti-RET multikinase inhibitors
**1.85**	4.08	4.77E-02	19	*HSD17B14*	Hydroxysteroid 17-beta dehydrogenase 14

Supplementary_Figure 1. Analytical structure

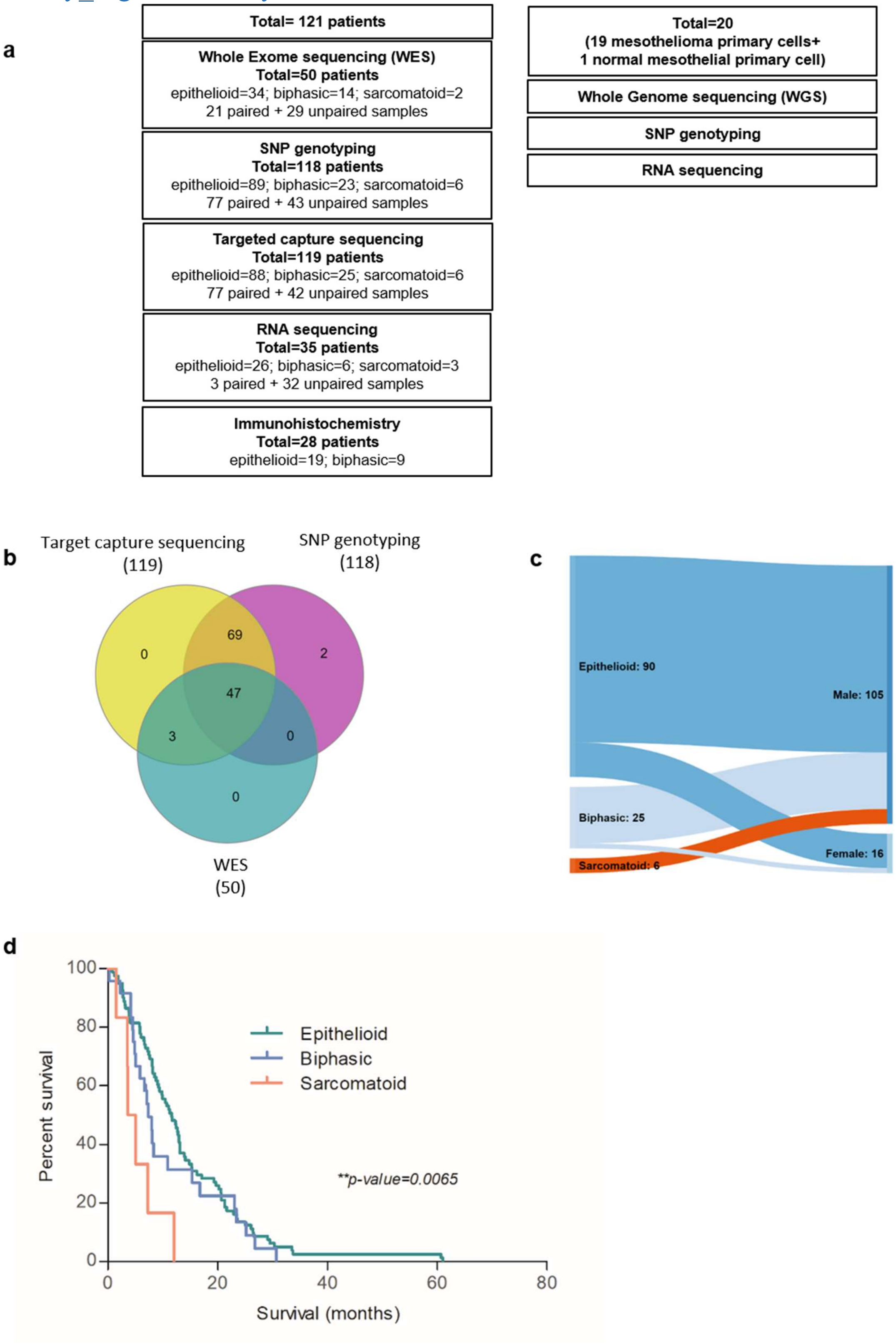

a) Summary of samples used in each analysis; b) Venn diagram showing overlapping of samples used in WES, SNP genotyping and targeted capture sequencing (please refer to Supplementary File 2- Table1 for further details); c) Sankey diagram on histological subtype and patients` gender; d) Kaplan-Meier survival curves on histological subtypes

Supplementary_Figure 2. Oncoplot from targeted sequencing panel

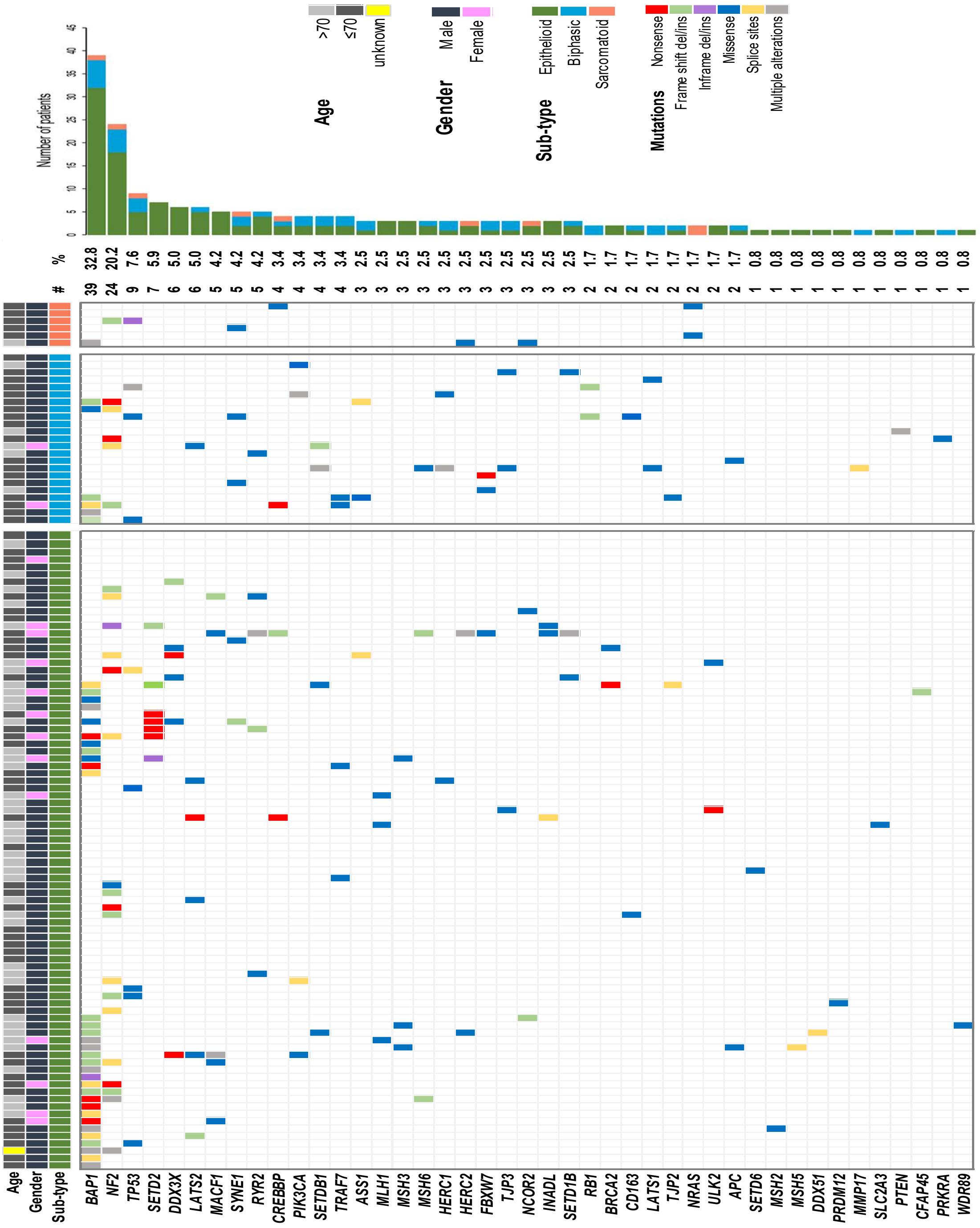

Oncoplot summarizing results from the targeted capture gene panel sequencing. Genes are arranged at the bottom from the most frequently mutated to the least. Three panels shown on the left-hand side represent histologic subtype (epithelioid, biphasic and sarcomatoid) age and gender. The bar plot on the top, shows the number of patients found to harbour mutation in the respective gene.

Supplementary_Figure 3. Mutation characteristics

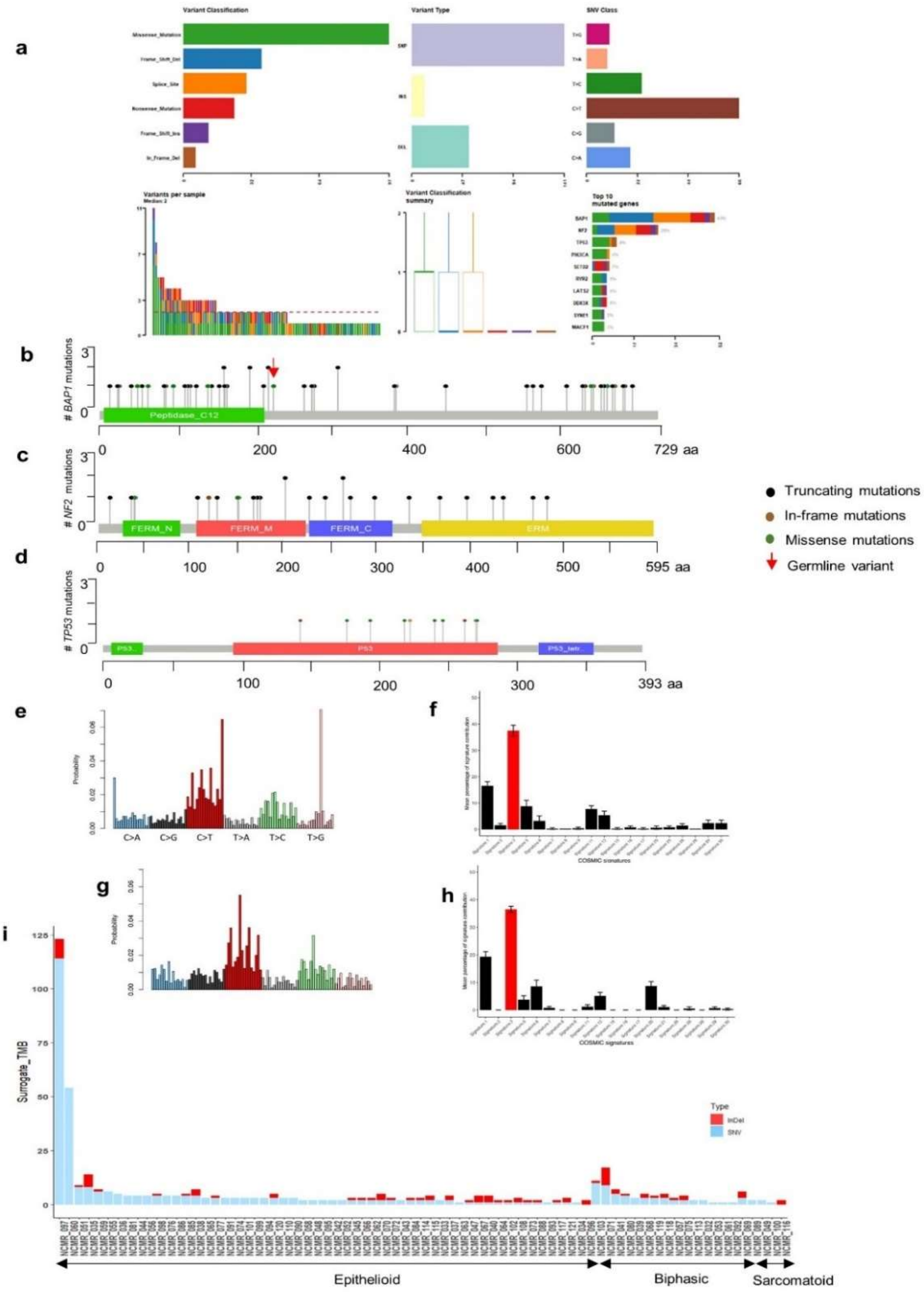

a) Summary of mutation spectrum observed for genes from targeted capture sequencing panel; b-d) Distribution of mutations in *BAP1*, *NF2*, and *TP53*; e) Mutational signature in 21 paired mesothelioma samples analysed by WES; f) Mean percentage contribution for COSMIC signatures in the WES: red bars indicate signatures associated with DNA damage; g) Mutational signature in 19 patient-derived PM cell lines from WGS; h) Mean percentage for COSMIC signatures in each cell line; i) Tumour mutation burden (TMB) derived from targeted capture sequencing of 57-gene panel and hence abbreviated as ‘Surrogate TMB’, in 77 paired samples. Briefly, all somatic SNVs or, InDels observed per sample, across the gene-panel are summed to derive surrogate TMB.

Supplementary_Figure 4. WES and RNA sequencing correlations for *RASSF7*, *RB1* and *SUFU* in primary and replication datasets

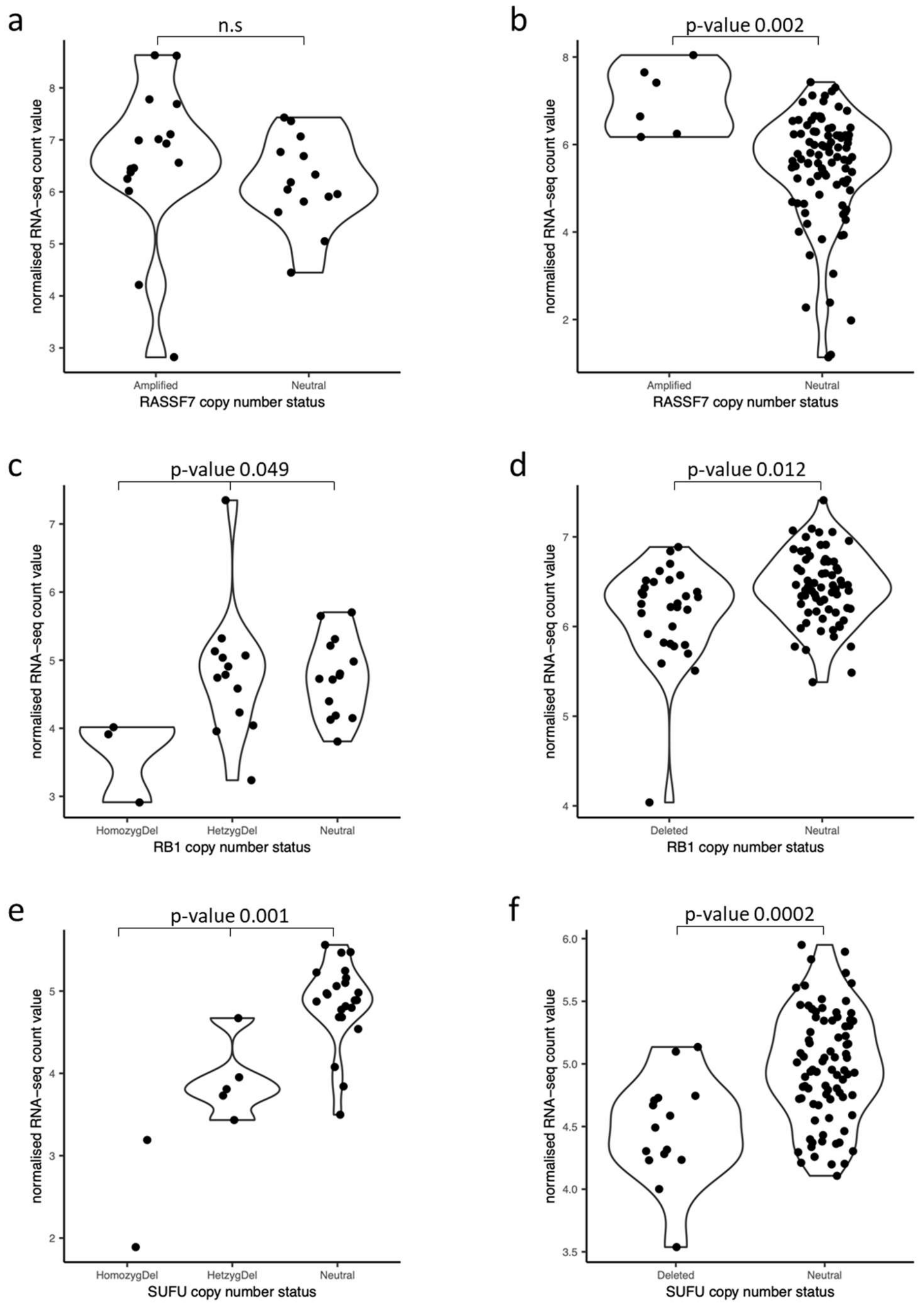

Copy-number profiles from SNP array (NCMR, n=30) (panels a, c and e) or WES (Bueno *et al*., n=98) (b, d and f). were correlated with gene expression from RNA-sequencing. Homozygous vs. heterozygous deletions of *RB1* and *SUFU* loci could be predicted from SNP array data, but we could not discriminate homozygous vs. heterozygous amplification of *RASSF7*. In the Bueno *et al.* WES data only presence or absence of CNAs could be determined. Kruskal-Wallis tests were applied to compare expression differences between three classes, and two classes were compared by Mann-Whitney tests. In each case the Bueno *et al*. results confirm the presence of correlations between CNA status and transcript abundance.

Supplementary_Figure 5. Oncoplot for WGS of mesothelioma primary cells

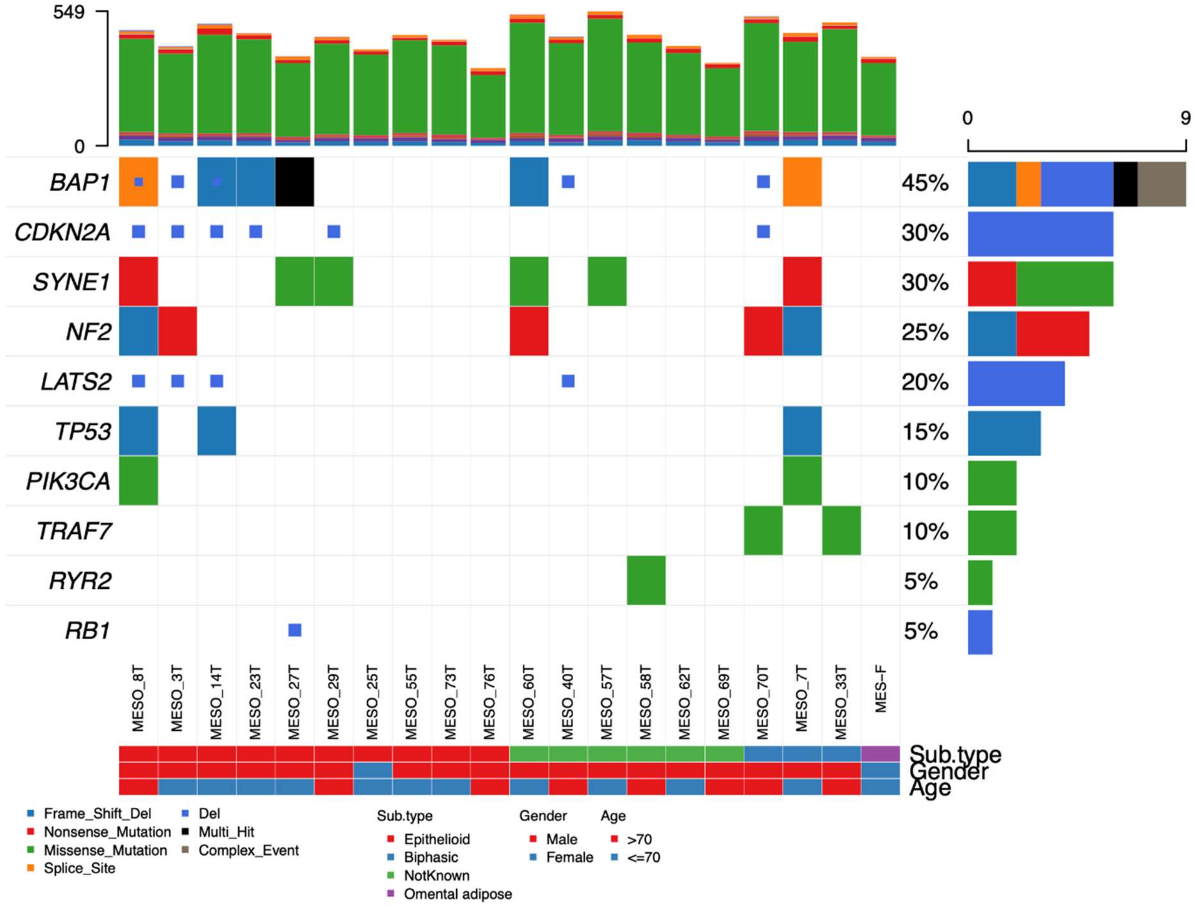

Oncoplot showing alterations in mesothelioma in whole-genome sequenced primary cell lines (19 mesothelioma and one mesothelial primary cell).

Supplementary_Figure 6. Effect of mutations in DNA damage repair related genes

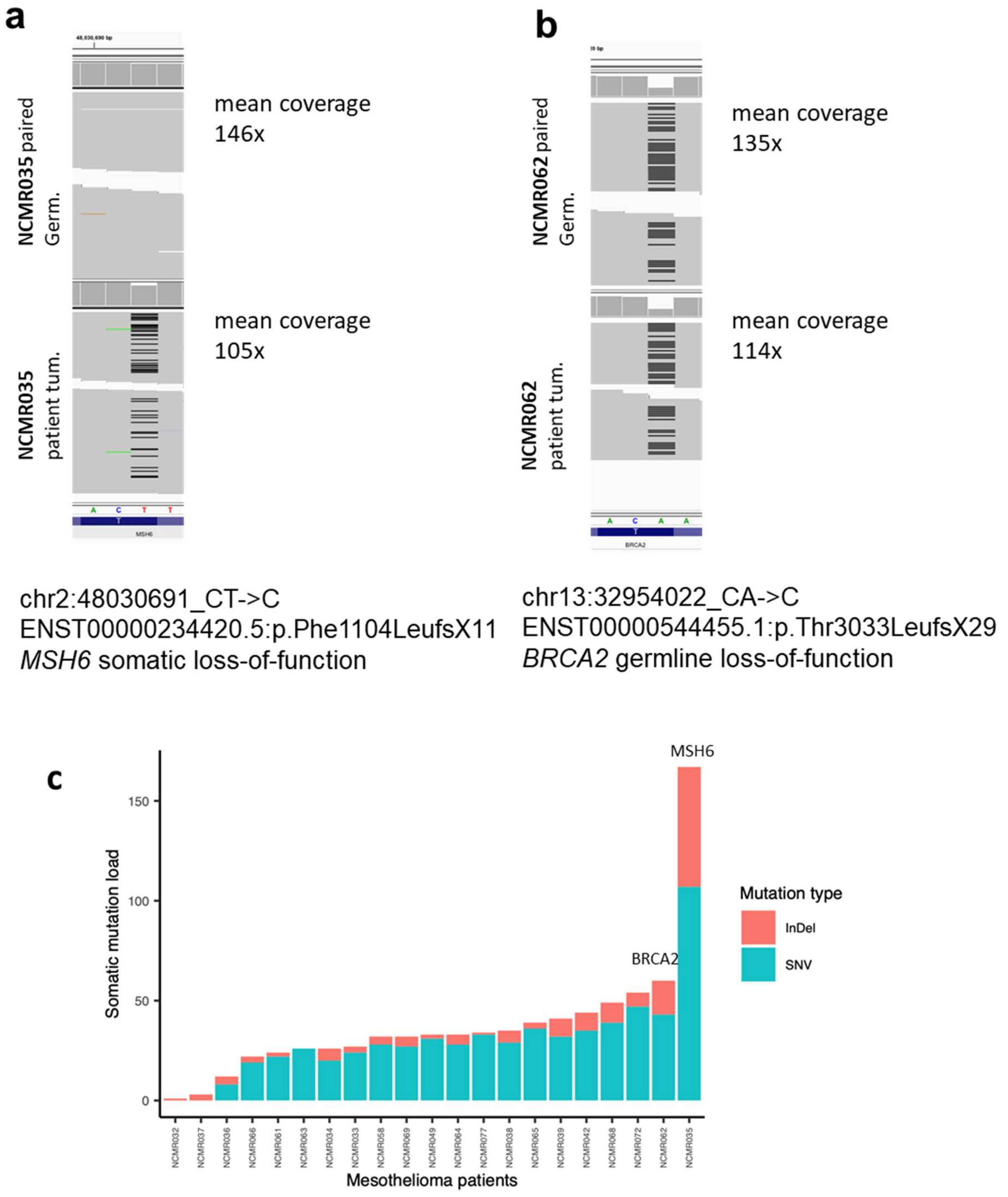

a and b) IGV (Integrated Genome Viewer) snapshots of matching genomic positions in patient tumour and the paired germline whole-exome sequencing (WES) BAM files. Upper panel shows coverage for the variant locus in the germline and the lower panel shows coverage for the same in the paired tumour sample. a) is from a patient with somatic MSH6 loss and b) is from a patient with germline BRCA2 loss. c) Somatic mutational load in tumours undergoing WES: BRCA2 and MHS6 mutations are at the extreme of the load spectrum

Supplementary_Figure 7. Response of mesothelioma primary cells to tested drugs.

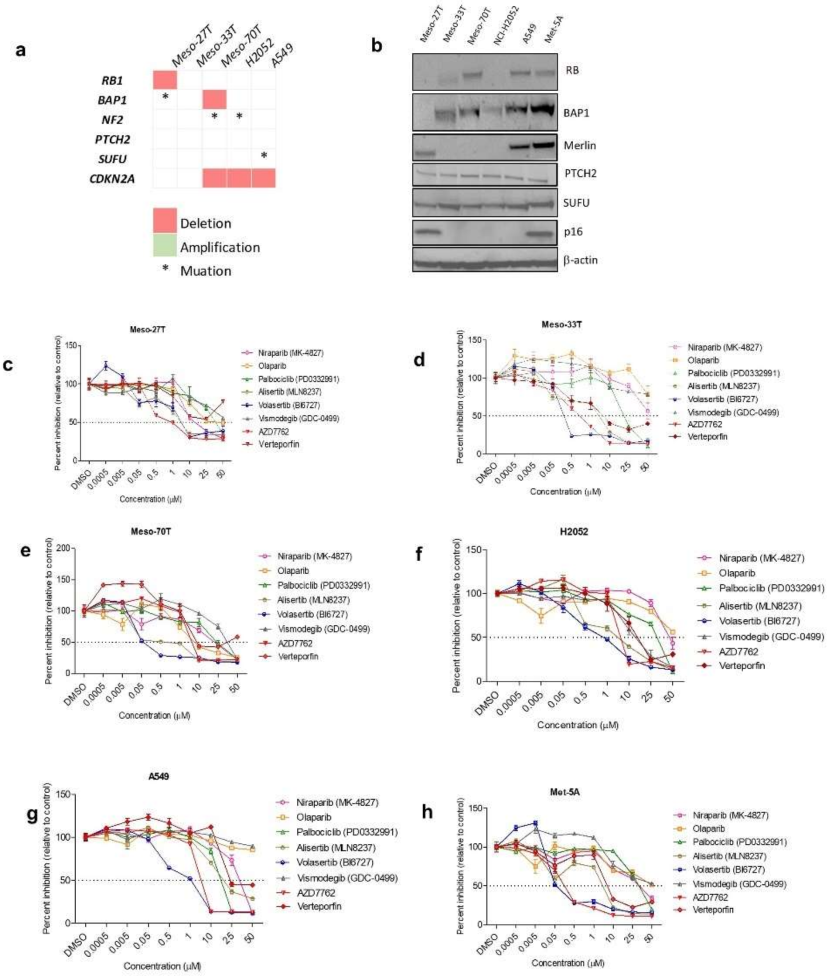

a) Schematic representation showing mutations and copy-number alterations status in the analysed mesothelioma primary cells and (commercially available) cell lines; b) Characterisation by Western blot of endogenous levels of RB, BAP1, Merlin (NF2), PTCH2, SUFU and p16 (CDKN2A) in primary cells and cell lines (original, unprocessed and uncropped blots are shown in Supplementary Figure 8); c-h) Sensitivity dose curves for patient-derived PM primary cells and cell lines against eight compounds. Standard errors of the mean are shown as bars. Dashed line mark indicates 50% inhibition by the drug

Supplementary_Figure 8. Original, uncropped and unprocessed blot images

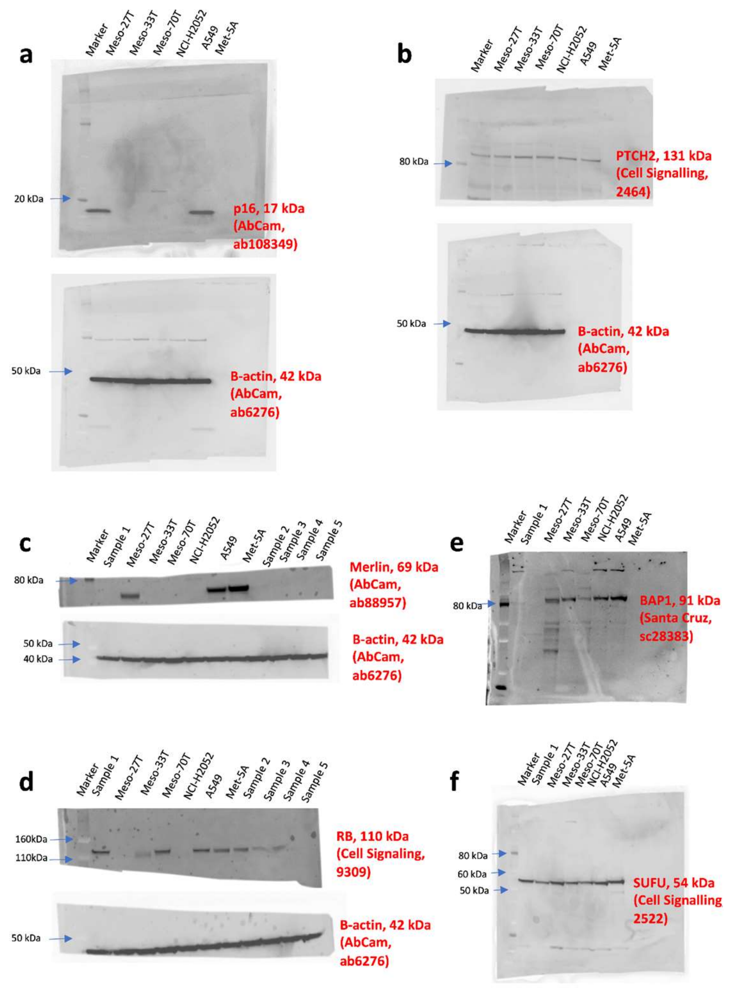

Western blot for a) p16 (upper image) and for beta-actin (bottom image). Same full-length blot was probed first for p16, then stripped (with Restore Stripping Buffer, Thermo Scientific) and probed for beta-actin; b) PTCH2 (upper image) and beta-actin (bottom image). Two PTCH2 antibodies were tested. Same full-length blot was probed first for PTCH2 (first antibody, failed), then stripped (with Restore Stripping Buffer, Thermo Scientific) and probed for beta-actin. Different blot sizes are due to blot (upper image) being cut to the desired size before second stripping and probing with the second PTCH2 antibody (shown in the image); c) Merlin (upper image) and beta-actin (bottom image). The full-length blot was first cut to the desired sizes before being probed with specified antibodies; d) RB (upper image) and beta-actin (bottom image). The full-length blot was first cut to the desired sizes before being probed with specified antibodies; e) BAP1 (beta-actin blot not included); f) SUFU (beta-actin blot not included).

Samples indicated as Sample 1-5 in panel c and d or Sample 1 in panel e and f are neither included in this article nor in Supplementary Figure 7b. Marker used – Novex Sharp pre-stained protein standard (Thermo Fischer Scientific).

In Supplementary Figure 7b, a representative image of beta-action is shown.
